# The dorsoventral patterning of *Musca domestica* embryos: insights into BMP/Dpp evolution from the base of the lower cyclorraphan flies

**DOI:** 10.1186/s13227-018-0102-5

**Published:** 2018-05-16

**Authors:** Christian Hodar, Verónica Cambiazo

**Affiliations:** 1 Laboratorio de Bioinformática y Expresión Génica, INTA-Universidad de Chile, El Líbano 5524, Santiago, Chile; 2 Fondap Center for Genome Regulation (CGR), Santiago, Chile

## Abstract

**Background:**

In the last few years, accumulated information has indicated that the evolution of an extra-embryonic membrane in dipterans was accompanied by changes in the gene regulatory network controlled by the BMP/Dpp pathway, which is responsible for dorsal patterning in these insects. However, only comparative analysis of gene expression levels between distant species with two extra-embryonic membranes, like *A. gambiae* or *C. albipunctata*, and *D. melanogaster,* has been conducted. Analysis of gene expression in ancestral species, which evolved closer to the amnioserosa origin, could provide new insights into the evolution of dorsoventral patterning in dipterans.

**Results:**

Here we describe the spatial expression of several key and downstream elements of the Dpp pathway and show the compared patterns of expression between *Musca* and *Drosophila* embryos, both dipterans with amnioserosa. Most of the analyzed gene showed a high degree of expression conservation, however, we found several differences in the gene expression pattern of *M. domestica* orthologs for *sog* and *tolloid*. Bioinformatics analysis of the promoter of both genes indicated that the variations could be related to the gain of several binding sites for the transcriptional factor Dorsal in the *Md.tld* promoter and Snail in the *Md.sog enhancer*. These altered expressions could explain the unclear formation of the pMad gradient in the *M. domestica* embryo, compared to the formation of the gradient in *D. melanogaster.*

**Conclusion:**

Gene expression changes during the dorsal–ventral patterning in insects contribute to the differentiation of extra-embryonic tissues as a consequence of changes in the gene regulatory network controlled by BMP/Dpp. In this work, in early *M. domestica* embryos, we identified the expression pattern of several genes members involved in the dorsoventral specification of the embryo. We believe that these data can contribute to understanding the evolution of the BMP/Dpp pathway, the regulation of BMP ligands, and the formation of a Dpp gradient in higher cyclorraphan flies.

**Electronic supplementary material:**

The online version of this article (10.1186/s13227-018-0102-5) contains supplementary material, which is available to authorized users.

## Background

Changes in the spatial pattern of gene expression and the gene regulatory mechanisms underlying variation between species have been studied [1–3] to understand how changes in gene regulation and expression cause adaptation and evolution [4, 5].

**Fig. 1:**
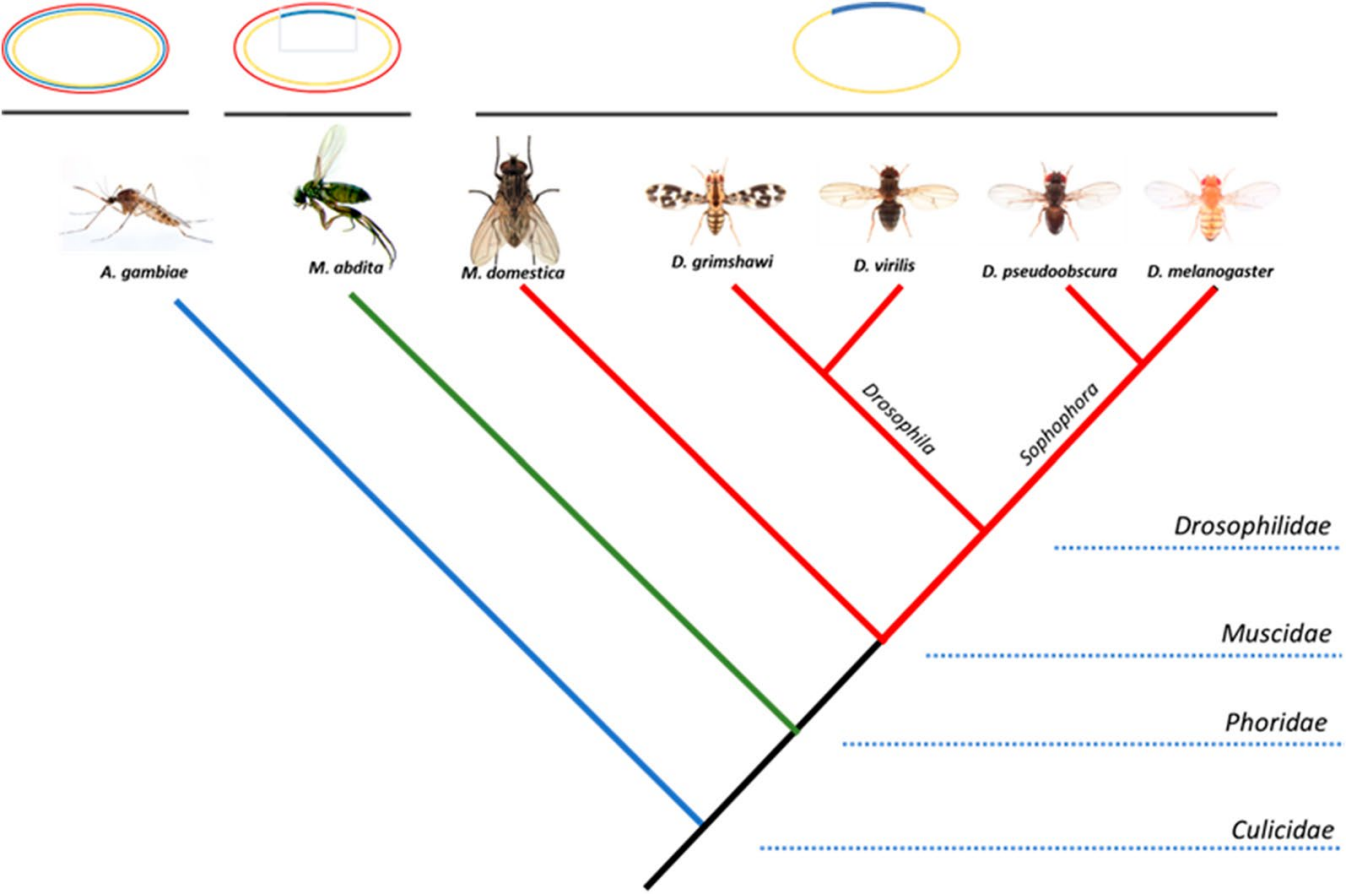
Schematic comparison of extra-embryonic tissues in dipteran insects. In *A. gambiae* (*Culicidae*), embryo (yellow) is surrounded by two extra-embryonic membranes, amnio (blue) and serosa (red). In lower cyclorraphan flies, as *M. abdita* (*Phoridae*), the serosa still covers the whole embryo, but the amnio is located dorsally. In higher cyclorraphan flies, like *M. domestica* or *D. melanogaster*, a unique tissue, the amnioserosa (dark blue), is develop in the dorsal domain of the embryo

A well-documented example of how changes in gene expression impact the phenotype comes from studies on the evolution of extra-embryonic tissues in dipterans. The embryos of most insect species are surrounded by two extra-embryonic tissues that are essential for development [6]: an outer membrane, the serosa, which completely covers the embryo and the yolk, and an internal amnion which in turn covers the ventral region of the embryo, delimiting the amniotic cavity. During the evolution of the order *Diptera*, the serosa and the amniotic cavity decreased in size and both epitheliums are differentiated from dorsal precursor cells [7]. This morphology is conserved in mosquitoes like *Anopheles gambiae* [8], but underwent a further variation in higher *Diptera*—in lower cyclorrhaphan flies such as *Megaselia abdita* and *Episyrphus balteatus*, the amnion is restricted to the dorsal side of the embryo and the serosa expands to the ventral embryonic regions [9, 10], while in higher cyclorrhaphan flies, such as *Drosophila melanogaster*, both epitheliums merged in a single extra-embryonic epithelium, the amnioserosa, which covers only the dorsal portion of the embryo during gastrulation [11] (Fig. 1). These major morphological changes are proposed to be correlated with several variations in the BMP/Dpp signaling pathway that controls extra-embryonic tissue specification in *Dipteran* embryos [12, 13].

A well-known example of a lineage-specific change in the BMP/Dpp signaling network came from the study of the evolution of the *screw* (Scw), *D. melanogaster* member of TGF-β family. Dpp and Scw form an extracellular activity gradient which patterns the dorsal ectoderm of the embryo into amnioserosa and dorsal epidermis [14]. Scw originated from a duplication of the BMP5/6/7/8 orthologue *Glass bottom boat* (*Gbb*) in the lineage leading to higher *Diptera* [15]. It is absent in the genomes of the moth *C. albipunctata* or the mosquito *A. gambiae*, suggesting that Scw signaling may have evolved specially for the specification of amnioserosa [16]. Conversely, in *A. gambiae* embryos, an increase in the dorsal expression domain of phosphorylated Mad (pMad), the signal transducer of the BMP/Dpp pathway, appears to be one of the causes of the expansion of the dorsal ectoderm and dorsal specification of the serosa in this species [8]. In contrast, a sharp peak of pMad expression in the dorsal-most cells of *D. melanogaster* embryos denotes the narrow domain of high BMP/Dpp activity that is required to differentiate the amnioserosa [17]. This high BMP/Dpp activity is essential for the expression of *zerknüllt* (*zen*), a homeobox gene, which is necessary for all aspects of amnioserosa development [13]. Changes in the expression pattern of *zen* have been associated with morphological changes in the extra-embryonic tissues in *Dipteran* embryos. Together with zen findings, it has been documented that changes in the BMP activity, during the blastula to gastrula transition, in distinct insects generate morphological variations of the extra-embryonic tissues [18]. This has been particularly studied comparing *D. melanogaster* with a basal species of cyclorrhaphan flies, *Megaselia abdita*, where a temporally differential control of the positive feedback for BMP refining occurs during blastoderm and gastrulation, associated with an ancestral expression of the ligand *eiger* in the cyclorraphan flies [18, 19]. Thus, different lines of evidence indicate that the evolutionary transition from two to one extra-embryonic membrane was accompanied by changes in the gene regulatory network controlled by the BMP/Dpp. More recent studies on Dpp pathway regulators and targets in the moth midge *C. albipunctata* suggested that a drift mechanism acts during development such that the rewiring of dorsal–ventral gene regulatory networks during the early patterning of embryos does not affect the expression pattern of the downstream signaling elements [20].

In this work, we aimed to characterize the gene expression pattern of primary regulators and downstream genes of the Dpp signaling pathway in embryos of *Musca domestica*, a higher Cyclorrhaphan fly, which, like *D. melanogaster*, develops a single extra-embryonic membrane. Although the morphology and early embryology of *M. domestica* and *D. melanogaster* are quite similar [21], these two flies are evolutionarily separated by at least 100 million years [22]. Since the origin of amnioserosa has been estimated to have occurred approximately 120 million years ago, the analysis of BMP/Dpp pathway components in *M. domestica* embryos may contribute to understanding whether the differences in dorsal patterning led to amnioserosa development. This will allow understand whether changes in BMP pathway, for example, between *D. melanogaster* and *M. abdita*, represent changes that occurred after the origin of the amnioserosa, in the base of these groups of flies, which have so far been described only in *D. melanogaster.*

## Methods

### Fly culture and embryo collection

Live larvae specimens of *M. domestica* were acquired from the Carolina Biological Supply Company and fed with an artificial wet diet (Carolina Biological Supply Company item #144,424), in a dark chamber at 26 °C. Adults were grown at 26 °C under a 16-/8-h light–day cycle and fed with granulated sugar and moist wood shavings as a water source. In order to stimulate fly oviposition, petri dishes containing wet cat food were introduced in the adult’s cages. For in situ hybridization of *M. domestica*, embryos were collected using a saline buffer (SB: 0.7% NaCl, 0.03% Triton X-100), dechorionized for 5 min. in 1:1 NaOCl/SB, and washed and fixed for 1 h in a 1:1 heptane and fixative solution (FS: 100 mM NaCl, 9.4% formaldehyde, 50 mM MgCl_2_, 50 mM EGTA, 100 mM Tris pH 9, 0.1% Tween-20). Finally, embryos were washed thrice in 100% methanol and four times in 100% ethanol and stored at − 20°. In situ hybridizations of *D. melanogaster* embryos were conducted as described in [23].

### Orthologs identification

Proteinortho tools [24] were used to identify groups of orthologs among the annotated proteins of *M. domestica*, version A1.1 (available at https://www.vectorbase.org/organisms/musca-domestica), three species from the *Drosophilidae* family, *D. melanogaster*, *D. pseudoobscura*, and *D. virilis*, and two species from the *Culicidae* family, *A. aegypti* and *A. gambiae*. Since Proteinortho is based on the blast similarity search and our outgroup is very divergent with *D. melanogaster*, the orthologs search was locally carried out using three combinations of coverage and identity for the match sequences—20%/20%, 15%/25%, and 15%/30% and fixing the blast cutoff e value at 1e−05 and a minimal 70% of similarity for additional hits. To avoid loss of orthologs in the clustering step of the algorithm due to imperfect pairing in the reciprocal blast, groups of interest genes were manually curated and realigned by blastp, using full protein sequences. In those cases where more than one ortholog was identified in the group, aligned sequences were used to construct a phylogeny using Bayesian inference, through the MrBayes [25] plugin in Geneious software.

### RNA probe synthesis and in situ hybridization

*M. domestica* cDNA was synthesized from a pool of total RNA, extracted from early embryos collected between 2 and 4 h postovoposition. PCR amplification using specific primers for each target gene, with a T7 binding sequence (5′-gaaataatacgactcactatagggaga-3′), was added to the reverse primer. In vitro transcription of RNA probes was performed according to the manufacturer’s instructions, using Digoxigenin (DIG)-RNA labeling mix (Roche, Mannheim, Germany) and T7 RNA polymerase. In situ hybridizations of *Drosophila* and *M. domestica* were carried out essentially as described in [23, 26]. Sequences for the primers used to synthesize the probes are listed in the table below.

**Table:** 

Gene	Forward (5′–3′)	Reverse (5′–3′)
Md.Zen	acaccccaaattccacagcc	ggtgttggagcagcatttgg
Md.Sog	tgagcgtctccatgttgtcc	atcatcgggagcaaaaacgc
Md.Tolloid	tcaggccatatcgattggacg	tttcgaaaaggtgttgcggg
Md.Brinker	aaagcagcactactaccccg	gatgtgggagagatggtggg
Md.Vnd	aaacgcacaatcctttggcc	gccgcatggatgaaggagaa
Md.Snail	agcttcatccattgcctccg	gactgaggatccactgctgg
Md.Pannier	cacaaccctgtggagacgca	gccaagtcgttttgcaactt
Md.Tailup	atgcagcgtgcctaaaatgc	aggtgatgaatttgcgtt
Md.Doc	cggcaatgttgaggctaaat	gttggggagaccagggtaat

### Immunostaining of embryos

Embryos were fixed and treated as described in [26]. The primary antibody was polyclonal anti-Phospho-Smad 1/5 (Cell Signalling; 1:10), the secondary was Alexa Fluor 488 Goat Anti-Rabbit IgG (Invitrogen; 1:500), and nuclear staining was done with TO-PRO3 (Molecular Probes; 10 μM). Fluorescently labeled embryos were mounted in DAKO or 3:1 glycerol/PBS. Confocal images were collected using the Confocal Microscope C2+ (Nikon) and processed using NIS-Elements Microscope Imaging Software (Nikon) and FIJI image analysis software [12].

### Promoter analysis

In *D. melanogaster*, the silencer element of *tolloid* has been reported [27] as located within the intergenic region between *tolloid* and its upstream gene, *tolkin.* To proceed with the detection, we first identify the orthologs of both genes in all the analyzed species to determine whether the synteny among these species is conserved. Next, we extract the intergenic and first intron sequence using a progressive MAUVE aligner [28]. Finally, we use the described position weight matrix (PWM) for Dorsal (MA0022.1, MA0023.1) and Brinker (MA0213.1) transcription factors in *D. melanogaster*, available in the JASPAR database [29]. We use UGENE software v1.29 to find potential binding sites for the transcription factor in noncoding regions of *D. simulans*, *D. pseudoobscura*, *D. virilis*, and *M. domestica*. In the case of *sog*, similar analysis was applied using the Dorsal PWM and the matrix for transcription factor Snail (MA0086.1). Cluster of sites was identified using ClusterDraw 2 software [30]. Logos for the consensus binding sites were drawn using the WebLogo online tool [31].

## Results

### Orthologs identification of Dpp signaling pathway members in *M. domestica*

**Fig. 2:**
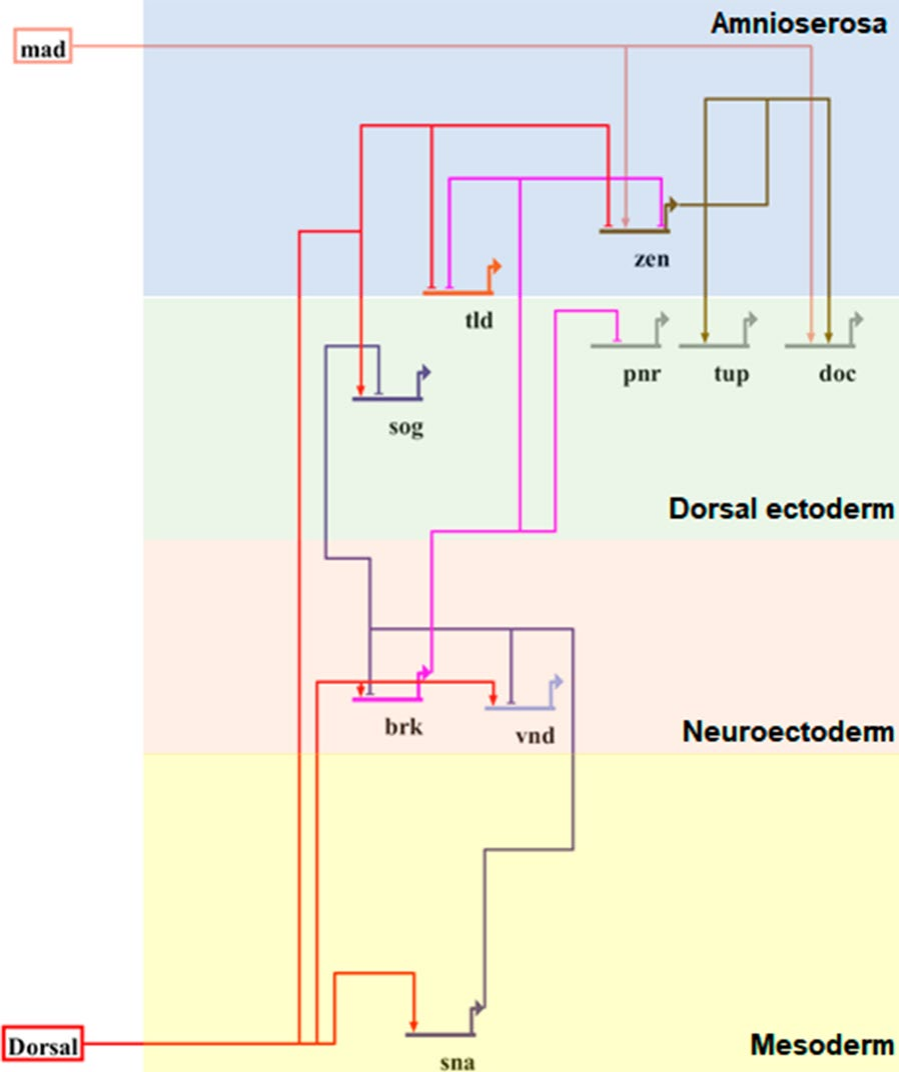
Schematic diagram with the members of the Dpp pathway examined in this work. The network is divided in colored boxes, based on the primary domains of expression of the genes. With the exception of transcription factor Dorsal, the expression of the remaining genes was examined by in situ hybridizations in embryos of *M. domestica* during cellularization stage

To describe the expression pattern of members of the Dpp pathway along the dorsoventral axis in the embryo of *M. domestica*, we first perform a search for orthologs, comparing the protein sequences from three species of the *Drosophilidae* family and *A. aegypti* and *A. gambiae*, both from *Culicidae* family, as the species outgroup, using Proteinortho software. With this analysis, we found a mean of 26,175 orthologs groups with a different number of members. An UPGMA analysis of the whole set of ortholog groups agreed with the position of *M. domestica* in the phylogeny of analyzed species (Additional file 1: Fig. S1). Within these groups of orthologs, we search for genes that have been previously characterized as members of the pathway in *D. melanogaster* (Table 1), with known expression in different domains of the *D. melanogaster* embryo during the stage of cellularization, to determine whether the expression in *M. domestica* is conserved (Fig. 2). Consequently, for eight of the analyzed genes, we detected a unique ortholog in the *M. domestica* genome. The two exceptions were *tolloid* and *Dorsocross*, in which more than one ortholog was found in their respective ortholog group—*tok* and *Dorsocross 2&3*. In these cases, phylogenetic reconstruction allows us to confirm the selection of a single ortholog to further characterize its expression pattern in *M. domestica* embryos (Additional file 1: Fig. S1).

**Table 1:** List of orthologs whose pattern of expression was analyzed in this work

Gene name	*M. domestica* genome ID	*D. melanogaster* ortholog	
*zerknullt* (Md.zen)	MDOA008087	FBgn0004053	CG1046
Mothers against dpp (Md.Mad)	MDOA012257	FBgn0011648	CG12399
Short gastrulation (Md.sog)	MDOA000243	FBgn0003463	CG9224
tolloid (Md.tld)	MDOA009895	FBgn0003719	CG6868
Brinker (Md.brk)	MDOA006277	FBgn0024250	CG9653
Ventral nervous system defective (Md.vnd)	MDOA009797	FBgn0261930	CG6172
Snail (Md.sna)	MDOA012986	FBgn0003448	CG3956
Pannier (Md.pnr)	MDOA006696	FBgn0003117	CG3978
Tailup (Md.tup)	MDOA012878	FBgn0003896	CG10619
Dorsocross 1 (Md.doc1)	MDOA015287	FBgn0028789	CG5133

### Comparative expression of the primary regulators of Dpp signaling pathway

First, we examined the distribution of the *M. domestica* orthologs in the two major regulators of dorsal patterning in the *D. melanogaster* embryo—the phosphorylated form of Mad (pMad), the final effector of the Dpp pathway, and the expression of the transcription factor *zerknüllt* (zen), which is responsible for all aspects of amnioserosa differentiation [32].

**Fig. 3:**
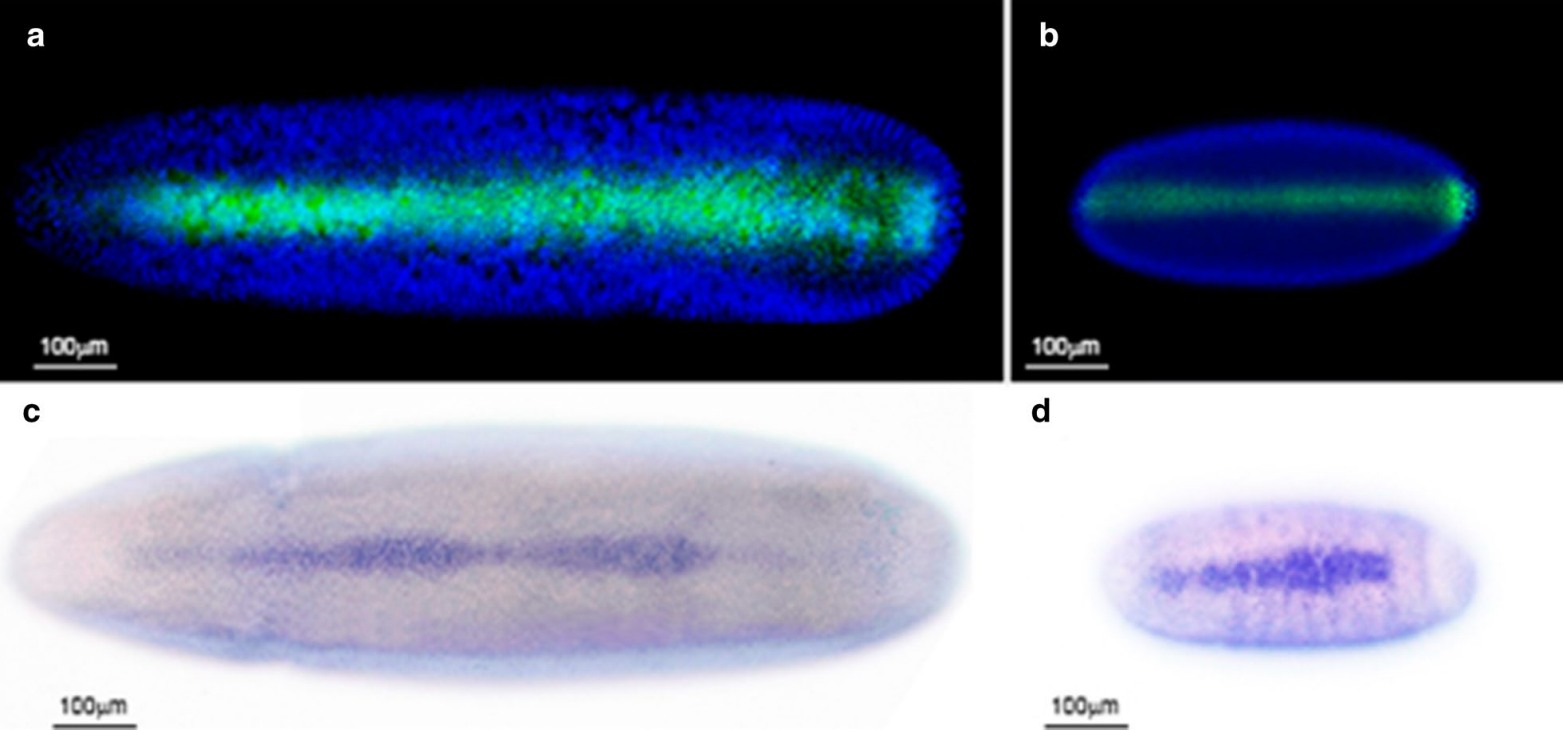
Comparative expression of primary regulators of Dpp in *D. melanogaster* and *M. domestica*. In the upper panel, we show the immunodetection of the phosphorylated form of Mad transcription factor (green) in dorsal views of stage 5 embryos of *M. domestica* (**a**) and *D melanogaster* (**b**). Blue signal corresponds to nuclei staining with TO-PRO. In the lower panel, we show the expression pattern of *zerknullt* (*zen*) mRNA during cellularization stages of *M. domestica* (**c**) and *D. melanogaster* (**d**) embryos, revealed by in situ hybridization. In all the cases, dorsal views of the embryos are oriented with the anterior region to the left and embryos pictures are on the same scale

During early embryogenesis in the cellular blastoderm stage, *M. domestica* pMad (*Md.pMad*) can be detected in a narrow domain at the dorsal midline (Fig. 3a), covering a longitudinal stripe from anterior to posterior pole, quite similar to the distribution observed in *D. melanogaster* embryos (Fig. 3b). For the *Md.zen* transcription factor, the transcript, where detected by in situ hybridization during mid-cellularization of the embryo in the dorsal midline cells, extended from the anterior pole to approximately two-thirds of the embryo. This expression is conserved between *M. domestica* and *D. melanogaster* (Fig. 3c, d). Furthermore, the dynamics of the expression of *Md.zen* are consistent with the described expression for *D. melanogaster*, where expression is repressed once amnioserosa is developed in the dorsal domain of the embryo, in contrast with the observed postgastrular expression in *M. abdita* [33] (Additional file 2: Fig. S2).

**Fig. 4:**
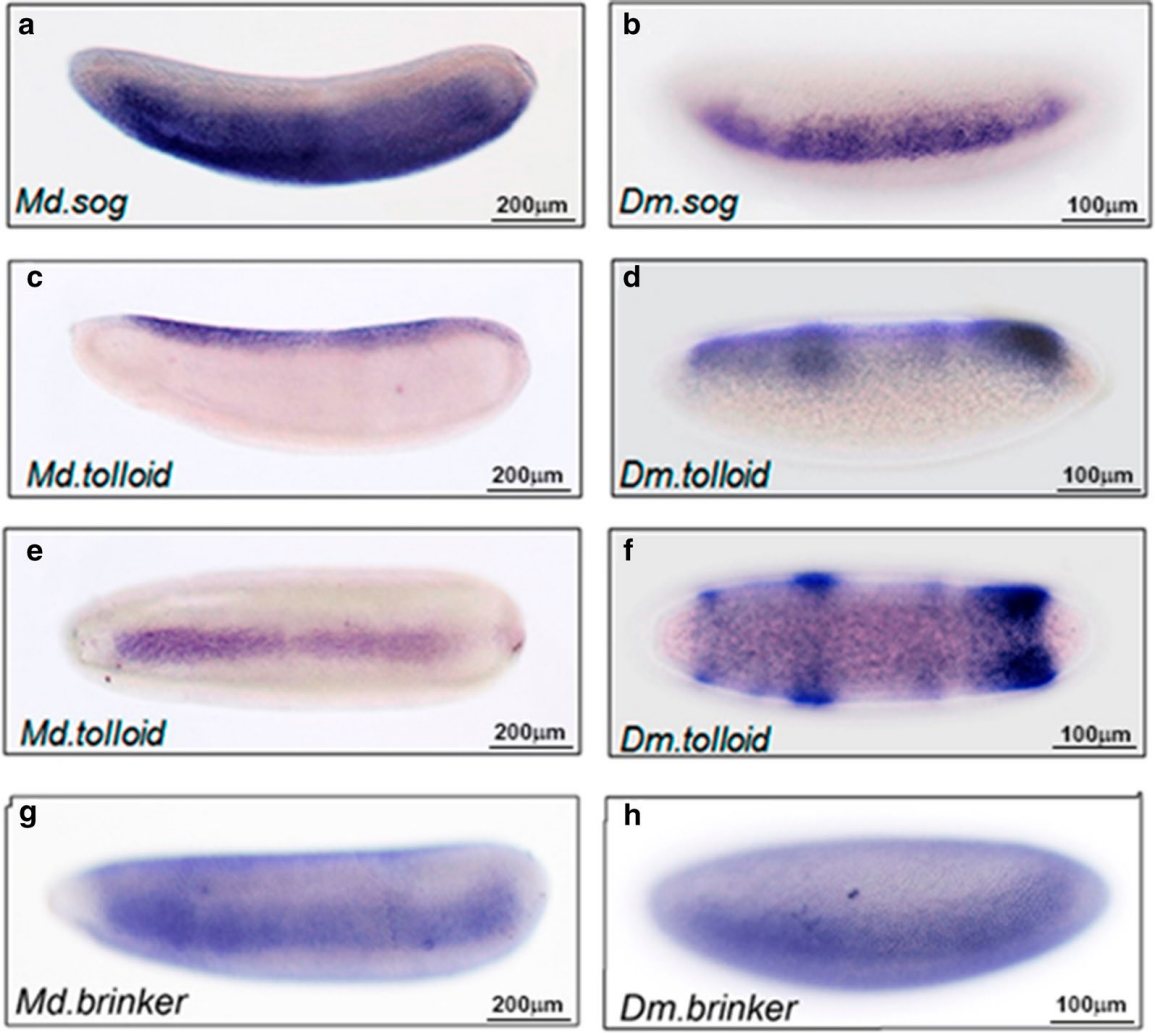
Comparative distribution of some key genes for the activation of the Dpp pathway. Images show the mRNA distribution, observed by in situ hybridization, during cellularization of *M. domestica* (**a**, **c**, **e**, **g**) and *D. melanogaster* embryos (**b**, **d**, **f**, **h**), revealed by in situ hybridization. In panels **a**–**d**, **g** and **h**, lateral views of the expression of *short gastrulation (sog)*, *tolloid* and *brinker* are shown. Panels **e**, **f** show a dorsal view of the expression of *tolloid* where differences in the amplitude of the pattern can be observed. In all the images, embryos are oriented with the anterior region to the left

We also examined the expression of the genes *short gastrulation* (*sog*) and *tolloid* (*tld*), whose products are required to shape the Dpp gradient [34]. The expression pattern of *sog*, the antagonist of the Dpp pathway in the embryo’s dorsal domains, is similar in both species along the anterior to posterior axis of the embryos. However, in *M. domestica*, the dorsal and ventral borders of *Md.sog* expression seemed to be slightly extended (Fig. 4a, b). Then, we examined the expression of the gene *tld* encoding a metalloprotease that cleaves *sog* at the Twisted-gastrulation (Tsg)-Sog-Dpp complexes to produce a peak of Dpp signaling along the dorsal midline of *D. melanogaster* embryos [35]. Interestingly, in the *M. domestica* embryo, *Md.tld* expression was restricted to the dorsal-most cells of the embryo, in a narrow stripe that resembled the distribution of pMad along the dorsal midline (Fig. 4c, e), unlike *D. melanogaster* embryos where expression of *tld* in the cellular blastoderm encompasses a broad dorsal domain (Fig. 4d, f). Interestingly, the dorsal expression of *Md.tolloid* remains dorsal during gastrulation (Additional file 3: Fig. S3). Additionally, we examined the expression of the transcriptional regulator *brinker* (*brk*) that sets the limits of the dorsal ectoderm in *D. melanogaster*. We found that in *M. domestica* the expression of *Md.brk* is located, from anterior to posterior, in lateral domains of the embryo, in a similar distribution to that observed in *D. melanogaster* (Fig. 4h).

### Expression pattern of *Md.vnd* and *Md.sna*

**Fig. 5:**
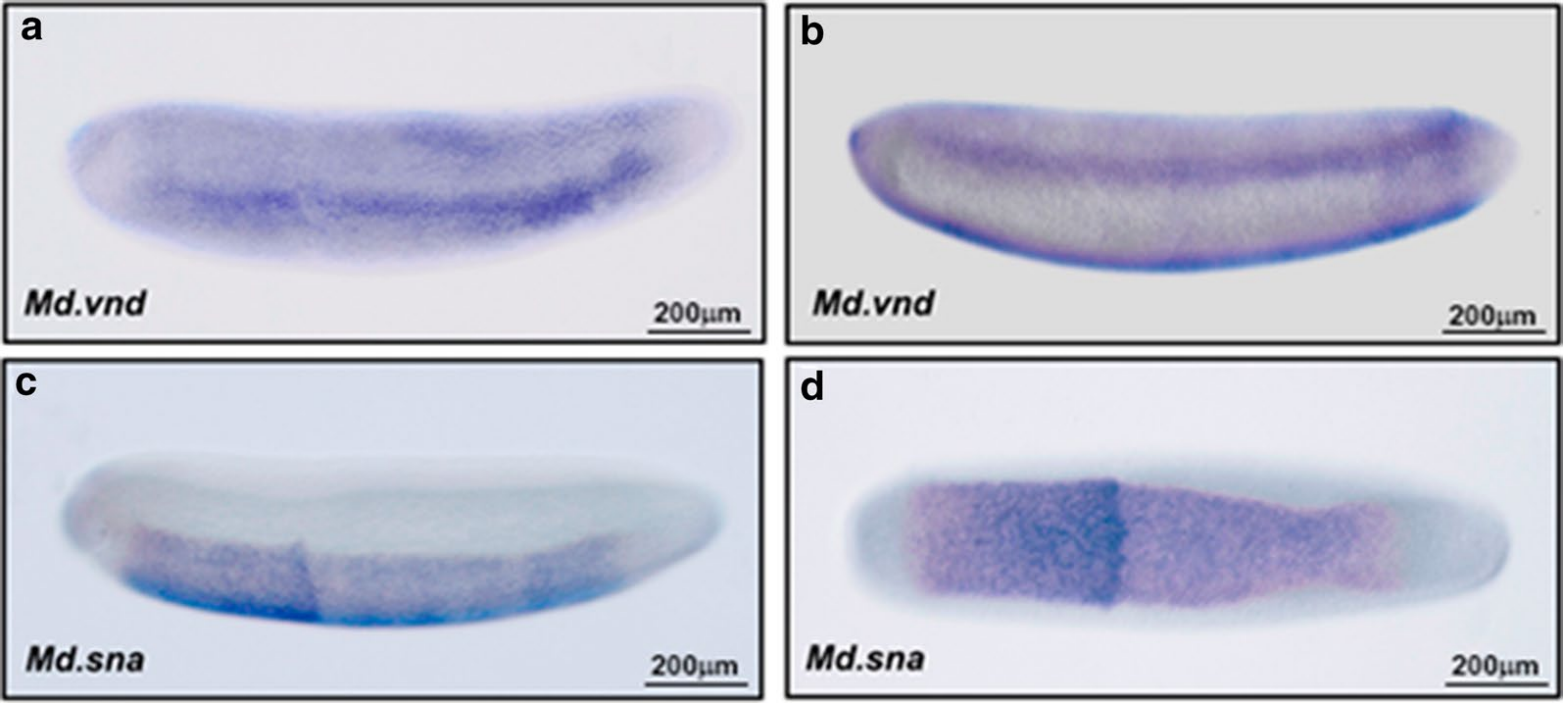
Comparative distribution for some ventral–lateral genes between *M. domestica* and *D. melanogaster.* Images show the mRNA distribution during cellularization stages, for *vnd* and *snail* transcripts, observed by in situ hybridization during cellularization of *M. domestica*. In panels **a**, **b**, lateral and ventral–lateral views of the expression of *ventral nervous system defective (vnd)* are shown. Panels **c**, **d**, show a lateral and dorsal view of the expression of *snail* transcription factor. In all the images, embryos are oriented with the anterior region to the right

In *D. melanogaster*, the expression of genes in the neuroectoderm is ventrally regulated by the action of a gradient of the Dorsal (Dl) transcription factor [36]. Based on the observed expression for *Md.sog* and *Md.tld*, we also examined the transcript distribution of *Md.vnd*, the ortholog of the *D. melanogaster* homeobox ventral nervous system defective (vnd) gene, which is expressed in the same domains as *sog* and *brk* during embryo cellularization [36]. We found that, during the same stage, *Md.vnd* expression is restricted to lateral stripes in the neuroectoderm domain and does not extend to dorsal or ventral regions (Fig. 5a, b), resembling the distribution detected for *Md.brk*. In *D. melanogaster*, the restricted expression of *vnd* or *brk* in ventrolateral domains of the embryo is induced by intermediate levels of *dl* [37]. Conversely, the transcription factor Snail is activated at high levels of Dl in ventral regions, acting as a ventral repressor for several genes, *sog* and *vnd* among them, by direct binding on their enhancers [38]. These findings suggest that the cause of extension of *Md.sog* expression into the embryo’s ventral domains is more related to a response to a loss of snail repression than variations in the gradient of the dorsal transcription factor. To corroborate this, we also examined, in *M. domestica*, the expression of the transcription factor *Md.snail* and found that its expression in the cellular blastoderm is restricted to the embryo’s ventral domain, similar to that in *D. melanogaster* (Fig. 5c, d), in response to high levels of Dl [37].

### *Cis* analysis of *Md.tld* and *Md.sog* regulatory sequences

**Fig. 6:**
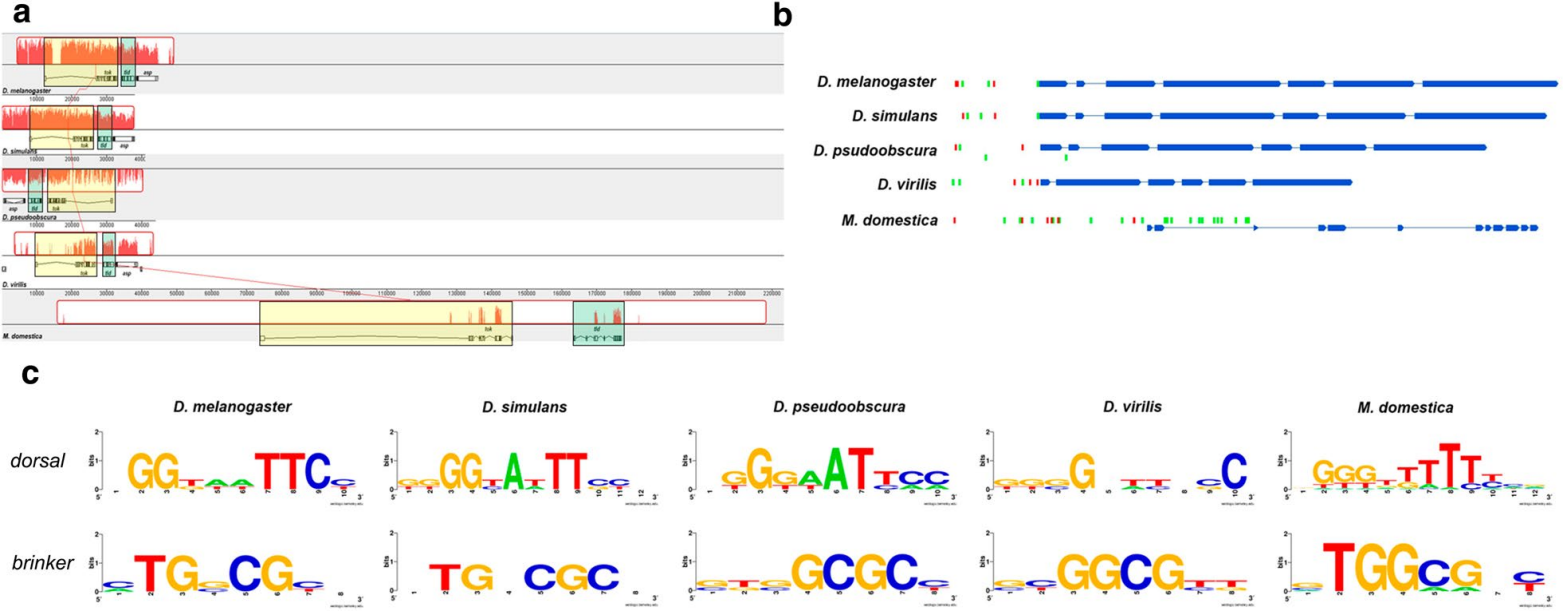
*Cis* comparative analysis of *tolloid* enhancer of *M. domestica*. The image in panel **a** shows the alignment of the genomic DNA close to *tolloid* gene in 4 species of *D. melanogaster* and *M. domestica*. Synteny was observed between the genes *tolkin* (yellow boxes) and *tolloid* (green boxes), and a red colored block in the first genome is connected by lines to similarly blocks in the remaining genomes. Image in panel **b** shows the unscaled organization of *tolloid* exons among the 5 species and the distribution of binding sites predicted for the transcriptions factors *dorsal* (green) and *brinker* (red). Panel **c** shows the logo representation for the binding sites identified in each analyzed species

The *tolloid* expression is regulated by the activity of *dorsal* gradient [27], which led us to hypothesize that restricted expression of *Md.tld* in the dorsal-most cells of the embryo can be explained by variations in the regulatory regions of the *tolloid* enhancer that allow the detection of low levels of nuclear *dorsal* transcription in more dorsolateral domains. In *D. melanogaster*, two of three binding sites for the *dorsal* transcription factor, within 800 bp fragment upstream from the first exon of the gene, can act as a silencer element for ventral domains in the embryo [27]. Using the public available position weight matrix (PWM) for the transcription factor *dorsal* in *D. melanogaster* [39], we conducted a search for *dorsal* binding sites in another species from the *Drosophila* clade. Using noncoding sequences from the 5′ upstream intergenic region of each *tolloid* ortholog (Fig. 6a), we detect three potential sites for the binding of *dorsal* in each *Drosophila* species; additionally, no binding sites were found in the first intron, except for *D. pseudoobscura* where one site was predicted (Fig. 6b). However, the same analysis for *M. domestica* identified eight potential binding sites in the intergenic region and 13 sites in the second intron. In *D. melanogaster*, *brinker* also acts as a repressor for *tolloid*, through one of three binding sites located in the same ventral repressor element described for *dorsal* binding sites [40]. Using the same strategy, we found at least two potential *brk* binding sites in *Drosophila*, while six potential binding sites were predicted in *M. domestica*. We believe that differences in the dorsolateral expression of *tolloid* could be related to an increased potential for binding repressors in the *M. domestica* silencer element, which results in the observed restricted domain of *Md.tld* expression.

**Fig. 7:**
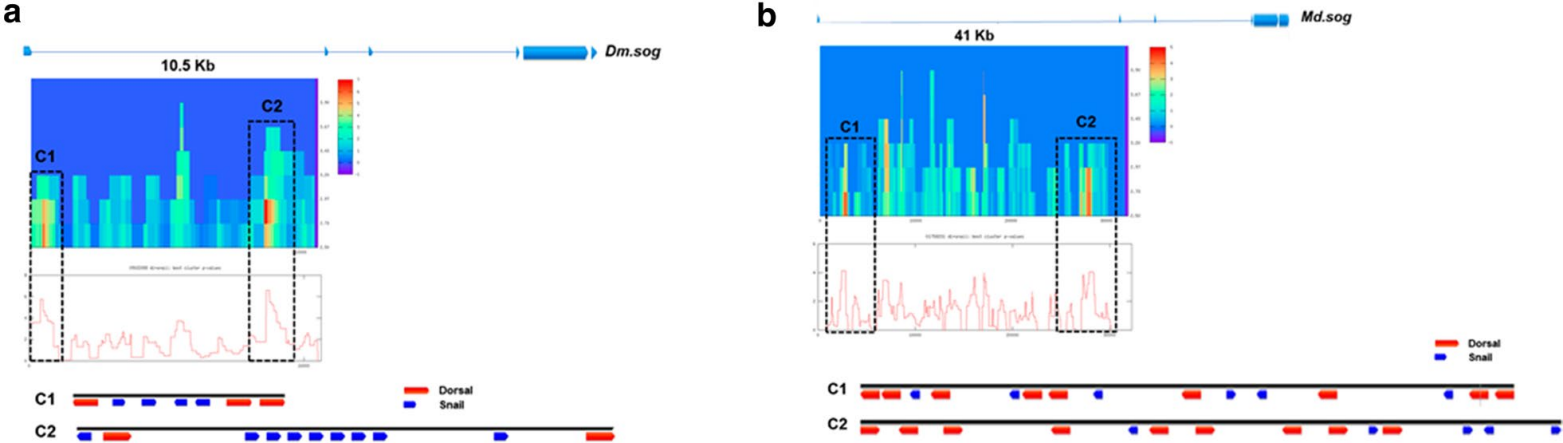
*Cis* comparative analysis of *sog* enhancer of *M. domestica*. The image shows the analysis performed with ClusterDraw to identified cluster of binding sites for the transcriptional factors Dorsal and Snail in the first intron of *D. melanogaster* (**a**) and *M. domestica* (**b**). *X*-axis of the graph indicates the space search, and *Y*-axis of the heat map indicates the match probabilities cutoff values and the density of the cluster is indicated by the color scale. Plots below show profiles for the best clusters *p* values, which allow the identification of significant clusters in each case (segmented boxes). In the bottom of **a**, **b** is shown the schematic distribution of potential binding sites for Dorsal (red) and Snail (blue) identified in each cluster

In *D. melanogaster,* the most ventral expression of *sog* is the resulting of two inputs: the response to the peak gradient of Dorsal, which in ventral domains of the embryo act as activator and the activity of transcriptional repressor Snail. Since *Md.sog* was detected into more ventral expression, in a similar pattern as in *A. gambiae* [8], we also analyze the differences between the regulatory domains of *Dm.sog* and *Md.sog* in order to compare the presence of both transcriptional factors. With the same strategy used for *Md.tolloid,* we search for putative binding sites for Dorsal and Snail in the first intron of *sog*, for which has been reported the presence of functional Dorsal binding sites in the *D. melanogaster* embryo. Our analysis identified several sites for Dorsal (*n* = 76) and Snail (*n* = 50) with different levels of confidentially. Since has been described that *sog* respond to low levels of the transcription factor in ventrolateral domains through a cluster of Dorsal binding sites, we use ClusterDraw 2 software and identified two clusters for *D. melanogaster* (Fig. 7a). The first one contains the already described sites for Dorsal (*n* = 3) and four sites for repressor Snail, while the second cluster contains two sites for Dorsal and 9 sites for Snail. Same analysis for *Md.sog* first intron identified similar number of sites for Dorsal (*n* = 69) and a large number for Dorsal (*n* = 227). Once these were clusterized, we identified two groups: both contain 9 sites for Dorsal binding, while for Snail we detect 6 and 5 sites in the first and second cluster respectively (Fig. 7b). We believe that this turnover of binding sites, between Dorsal and Snail, suggests that expansion of *Md.sog* expression into the most ventral part of the embryo could be drive by two effects: a lack of repression by Snail and the presence of increased sites for Dorsal acting as activator. This is consistent with the idea that in *D. melanogaster* regulatory regions for *sog* acquire new sites for binding Snail. In addition, despite the increased number of Dorsal binding sites in cluster 1 of *M. domestica*, we don't find the presence of T-rich domains five bases upstream Dorsal sites, which has been indicated as necessary to cause ventral repression in *D. melanogaster* [41]. In contrast, in *M. domestica*, T-rich sequences appear to be more distal from the Dorsal binding sites.

### Comparative expression of Dpp pathway downstream genes

**Fig. 8:**
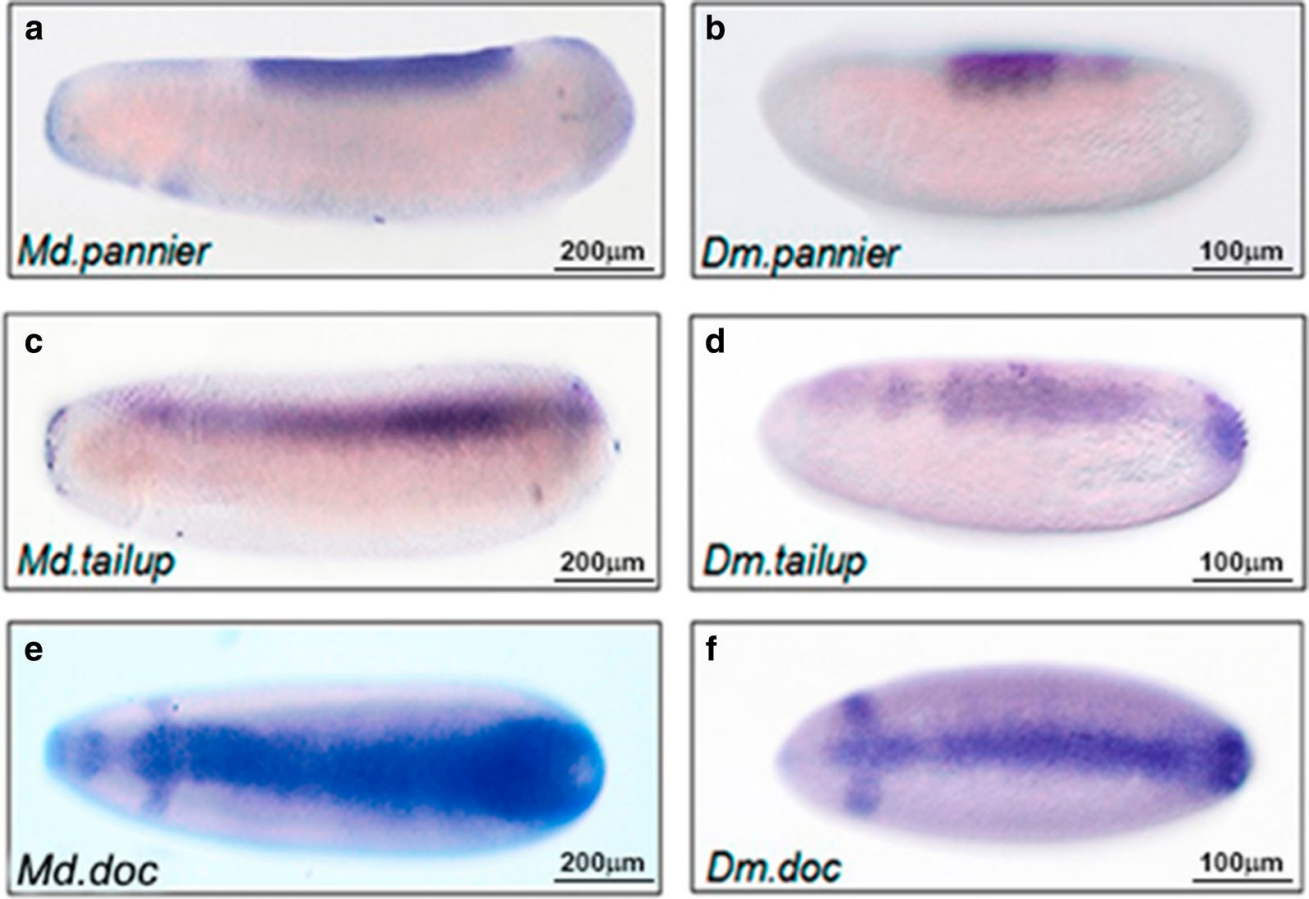
Comparative distribution for some *U*-*shaped* genes, between *M. domestica* and *D. melanogaster.* Images show the distribution of mRNA during cellularization stages, for some members of the *U*-shaped group of genes, between *M. domestica* (**a**, **c**, **e**) and *D. melanogaster* (**b**, **d**, **f**) embryos, revealed by in situ hybridization. In panels **a**–**d**, lateral views of the expression of *pannier* and *tailup* are shown. In panels **e**, **f**, dorsal view of *Dorsocross* is shown. In all the images, embryos are oriented with the anterior region to the left

Next, we examined the expression patterns of some members of the *u*-*shaped* group of genes, which are known target genes of the Dpp pathway. They are expressed in dorsal domains of early *D. melanogaster* embryos, encompassing a region that includes the presumptive amnioserosa and dorsal epidermis. All the *u*-*shaped* genes encode transcription factors, and different lines of evidence indicate that they are all involved in the maintenance of amnioserosa [42–44] and several other functions during embryo development. Among the u-shaped genes, *pannier* (*Md*.*pnr*) [45] displayed a temporal and spatial pattern similar to that observed in *D. melanogaster* embryos [46]. Thus, *pnr* mRNA was detected in a dorsal domain of early *M. domestica* embryos extending from 20 to 60% of the egg length (Fig. 8a, b). The expression of *tailup (Md.tup)*, which, in *D. melanogaster*, is required for the maintenance of amnioserosa, and posterior heart development [42] was detected along the dorsal midline of the embryo, encompassing the presumptive amnioserosa and the dorsal head (Fig. 8c). This expression pattern is similar to that observed in *D. melanogaster* (Fig. 8d). However, the stripes of expression of *Md.pnr* and *Md.tup* appear to be less variable in width and more restricted to the amnioserosa’s presumptive region than their counterparts in *D. melanogaster*. *Dorsocross*-*1 (doc1)*, a T-box transcription factor required for the proper patterning of dorsolateral ectoderm [43], was also detected along the dorsal midline of *M. domestica* embryos, including a dorsoanterior transverse stripe in the presumptive procephalic neuroectoderm, displaying a spatial pattern that is comparable to the expression observed in *D. melanogaster* (Fig. 8e, f).

## Discussion

It is well known that in *Dipteran* lineages, particularly in the *Drosophila* species group, the differentiation of the extra-embryonic tissues is controlled by a gene regulatory network, triggered by an extracellular gradient of the morphogen Decapentaplegic (Dpp). Our analysis indicates that the dorsal expression of primary regulators of this signaling pathway, transcription factors pMad and *Zen*, is conserved between *D. melanogaster* and *M. domestica*, during early embryo development, suggesting that their dorsal expression originated early in the higher cyclorrhaphan flies. We believe that this dorsal expression was begun almost concomitant with the development of the amnioserosa, early in the lineages of these flies, which suggests that the variations in the dorsal specification of extra-embryonic epitheliums, previously observed among *A. gambiae* and *D. melanogaster* [8] or *M. abdita* [19] start early in the lineage of these dipterans. We proved that the embryonic expression of downstream genes of the Dpp pathways, required for dorsal patterning and amnioserosa maintenance, was conserved between *M. domestica* and *D. melanogaster* embryos, contributing to support the previous proposal. However, two genes, *Md.sog* and *Md.tld*, showed differences in their spatial expression patterns between these species.

The expression of *Md.sog* observed during embryo cellularization is the only neuroectoderm gene examined here that reaches ventral domains. While in *D. melanogaster* or other *Drosophila* species [47], *sog* expression is detected in lateral stripes but excluded from ventral regions of the embryo, the *Md.sog* expression looks like the expanded expression of *sog* orthologs detected in *A. gambiae* and *C. albipunctata* embryos [8, 20]. The upper border expression of *Md.brk* and *Md.vnd* indicates that the neurogenic ectoderm domain is conserved during cellularization, and since *Md.snail* expression is also conserved between *D. melanogaster* and *M. domestica*, we suggest that the extended expression of *Md.sog* may be due to sequence-level changes in *Md.sog* enhancers [48], similar to that observed in *A. gambiae sog* enhancer [8], which suggest that ventral repression of *sog* evolves in the lineage of cyclorraphan flies.

Across the insects, the pattern of expression for BMP/Dpp ligands during cellularization stages or prior to gastrulation is quite divergent, from the two distinct anterior and posterior domains in the honeybee *A. mellifera* [49] to the ubiquitous expression in the *T. castaneum* embryo [50]. In the particular case of *Diptera*, in *A. gambiae*, a broader *dpp* expression corresponds to an expansion of the pMad activity into the dorsal ectoderm in the embryo, ventrally limited by the expression of *sog* [8]. A similar mechanism is found in *C. albipunctata* embryos, where pMad can be detected in the dorsal midline and extended to both poles of the embryo, but the ventral expression of *sog* delimits the boundary of *dpp* activity [20]. In *A. gambiae*, *tolloid* expression is restricted to lateral regions of the embryo and, together with ventral *sog*, contributes to limit the expansion of the Dpp into ectodermal domains [8]. *Md.tld* also shows a very divergent pattern of expression, characterized by a narrow domain of expression in the dorsal-most cells of the embryo, in contrast with *D. melanogaster*, where *tolloid* is detected in the dorsal side of the embryo during early cellularization and in the dorsal ectoderm during late cellularization and at the onset of gastrulation [51]. In *D. melanogaster*, this dynamic expression is initially regulated by the control of *dorsal* and then by the action of the transcriptional repressor *brinker*, which prevents the expression of *tolloid* in the neuroectoderm domain, resulting in a confined expression of *dpp* in dorsal domains of the embryo [52]. In consequence, low levels of pMad could be detected in a broad dorsal domain, and then this expression pattern is further refined to the dorsal-most cells by the action of secreted *tolloid* [53]. In *M. domestica* embryos, pMad did not show low levels of expression in the embryo’s dorsolateral domain, but rather an accumulation in its dorsal midline, suggesting that the observed dorsal expression of *Md.tld* may contribute to restricting the peak of pMad accumulation in the dorsal-most cells of the embryo. This might be attributable to an extension of the neuroectoderm into more dorsal regions; however, the conserved expression of *brk*, *vnd*, and *sna* between *D. melanogaster* and *M. domestica* leads us to suggest that the restricted expression of *Md.tld* in the dorsal-most cells of the *Md.tld* embryo might be due to repression of *Md.tld* expression in more lateral domains of the *M. domestica* embryo. Analysis of the regulatory regions of *Md.tld* leads us to propose that the accumulation of pMad in the dorsal midline of the *M. domestica* embryo during cellularization is the result of a *dorsal* repression in the embryo’s dorsolateral domains.

In *D. melanogaster*, once the gradient of pMad is formed and the peak of expression is reached in the embryos’ midline, the dorsal-most cells of the embryo acquire a final fate in the differentiation or maintenance of the amnioserosa [42]. We examined the expression of three genes which, in *D. melanogaster*, are required for the proper formation of the amnioserosa after gastrulation: *tailup* [42], *pannier* [54], and *Dorsocross* [43]. We observed that, in *M. domestica*, the expression patterns are conserved in the dorsal-most domain of the embryo, in a clear difference with the expression observed in dipterans with two extra-embryonic tissues. For example, orthologs of *tailup* and *Dorsocross* in *A. gambiae* are expressed only in the amnio lineage of dorsal ectoderm [8] and, similarly, *pannier* has been detected in the amnio cells in *M. abdita* [9]. In *D. melanogaster*, these three genes are essential for normal heart development [55], which suggests that, once amnioserosa evolves in the lineage of cyclorraphan flies, the expression pattern for these genes are rapidly restricted to amnioserosa in order to preserve their dorsal activity as key elements for heart development.

## Conclusion

The analysis of gene expression changes during the dorsal–ventral patterning of the mosquito *A. gambiae*, the lower Cyclorrhapha *M. abdita*, or the moth midge *C. albipunctata*, revealing that changes in the BMP/Dpp signaling pathway contribute to morphological variations in the differentiation of extra-embryonic tissues as a consequence of changes in the gene regulatory network controlled by BMP/Dpp [8, 20, 33], which evolves into the formation of the amnioserosa in the higher Cyclorrhaphan flies, such as *D. melanogaster*. In this work, in early embryos of *M. domestica*, we identified the expression pattern of several genes members involved in the dorsoventral specification of the embryo. Many of these genes have already been described in insects with two extra-embryonic epitheliums or in *D. melanogaster. M. domestica* and *D. melanogaster* shared a common ancestor approximately 100 million years ago, which is distant enough for major changes to occur, but since their morphology and early embryology are very similar, we believe that these data can contribute to understanding the evolution of the BMP/Dpp pathway and in particular, based on the expression of *Md.sog* and *Md.tld*, the regulation of BMP ligands, and the formation of a Dpp gradient in the higher cyclorraphan fly.

## Additional files

**Additional file 1: Fig.** **S1.** Panel (A) shows the UPGMA phylogenetic reconstruction based on the total number of orthologs identified by Proteinortho. Species that have a large part of their proteome in common are grouped. Panels (B) and (C) correspond to phylogenetics trees generated by bayesian approach, based on the alignment of the whole orthologs set for *tolloid* and *Dorsocross* proteins, respectively.

**Additional file 2: Fig.** **S2.** Images show the dynamics of the expression, revealed by in situ hybridization, of *Md.zen* during consecutive stages of embryo development: (A) early cellular blastoderm, (B) late cellular blastoderm at the onset of gastrulation, (C) during gastrula stage and (D) at the end of germ band extension. In all the cases, embryos are oriented from anterior to the left. All images correspond to lateral views except (B) which is a dorsal view.

**Additional file 3: Fig.** **S3.** Images show the dynamics of the expression, revealed by in situ hybridization, of *Md.tld* from (A) early cellular blastoderm, the onset of gastrulation (B) and during gastrula stage (C). In all the cases, embryos are oriented with anterior to the left. All images correspond to lateral views except (B) which is a lightly ventrolateral view.

## References

[1] Jimenez-Guri E, Huerta-Cepas J, Cozzuto L, Wotton KR, Kang H, Himmelbauer H, Roma G, Gabaldon T, Jaeger J (2013). Comparative transcriptomics of early dipteran development. BMC Genom.

[2] Merkin J, Russell C, Chen P, Burge CB (2012). Evolutionary dynamics of gene and isoform regulation in Mammalian tissues. Science.

[3] Silver DH, Levin M, Yanai I (2012). Identifying functional links between genes by evolutionary transcriptomics. Mol BioSyst.

[4] Battle A, Mostafavi S, Zhu X, Potash JB, Weissman MM, McCormick C, Haudenschild CD, Beckman KB, Shi J, Mei R (2014). Characterizing the genetic basis of transcriptome diversity through RNA-sequencing of 922 individuals. Genome Res.

[5] Jordan IK, Marino-Ramirez L, Koonin EV (2005). Evolutionary significance of gene expression divergence. Gene.

[6] Panfilio KA (2008). Extraembryonic development in insects and the acrobatics of blastokinesis. Dev Biol.

[7] van der Zee M, Berns N, Roth S (2005). Distinct functions of the *Tribolium zerknullt* genes in serosa specification and dorsal closure. Curr Biol CB.

[8] Goltsev Y, Fuse N, Frasch M, Zinzen RP, Lanzaro G, Levine M (2007). Evolution of the dorsal–ventral patterning network in the mosquito *Anopheles gambiae*. Development.

[9] Rafiqi AM, Lemke S, Ferguson S, Stauber M, Schmidt-Ott U (2008). Evolutionary origin of the amnioserosa in cyclorrhaphan flies correlates with spatial and temporal expression changes of zen. Proc Natl Acad Sci USA.

[10] Lemke S, Antonopoulos DA, Meyer F, Domanus MH, Schmidt-Ott U (2011). BMP signaling components in embryonic transcriptomes of the hover fly *Episyrphus balteatus* (Syrphidae). BMC Genom.

[11] Schmidt-Ott U (2000). The amnioserosa is an apomorphic character of cyclorrhaphan flies. Dev Genes Evol.

[12] Schindelin J, Arganda-Carreras I, Frise E, Kaynig V, Longair M, Pietzsch T, Preibisch S, Rueden C, Saalfeld S, Schmid B (2012). Fiji: an open-source platform for biological-image analysis. Nat Methods.

[13] Schmidt-Ott U, Rafiqi AM, Lemke S (2010). Hox3/zen and the evolution of extraembryonic epithelia in insects. Adv Exp Med Biol.

[14] Arora K, Levine MS, O’Connor MB (1994). The screw gene encodes a ubiquitously expressed member of the TGF-beta family required for specification of dorsal cell fates in the *Drosophila* embryo. Genes Dev.

[15] Van der Zee M, da Fonseca RN, Roth S (2008). TGFbeta signaling in *Tribolium*: vertebrate-like components in a beetle. Dev Genes Evol.

[16] Fritsch C, Lanfear R, Ray RP (2010). Rapid evolution of a novel signalling mechanism by concerted duplication and divergence of a BMP ligand and its extracellular modulators. Dev Genes Evol.

[17] Xu M, Kirov N, Rushlow C (2005). Peak levels of BMP in the *Drosophila* embryo control target genes by a feed-forward mechanism. Development.

[18] Rafiqi AM, Park CH, Kwan CW, Lemke S, Schmidt-Ott U (2012). BMP-dependent serosa and amnion specification in the scuttle fly *Megaselia abdita*. Development.

[19] Kwan CW, Gavin-Smyth J, Ferguson EL, Schmidt-Ott U (2016). Functional evolution of a morphogenetic gradient. eLife.

[20] Wotton KR, Alcaine-Colet A, Jaeger J, Jimenez-Guri E (2017). Non-canonical dorsoventral patterning in the moth midge *Clogmia albipunctata*. EvoDevo.

[21] Hewitt CG (1914). The house-fly, *Musca domestica* Linn: its structure, habits, development, relation to disease and control.

[22] Hennig W, Pont AC (1981). Insect phylogeny.

[23] Zuniga A, Hodar C, Hanna P, Ibanez F, Moreno P, Pulgar R, Pastenes L, Gonzalez M, Cambiazo V (2009). Genes encoding novel secreted and transmembrane proteins are temporally and spatially regulated during *Drosophila melanogaster* embryogenesis. BMC Biol.

[24] Lechner M, Findeiss S, Steiner L, Marz M, Stadler PF, Prohaska SJ (2011). Proteinortho: detection of (co-)orthologs in large-scale analysis. BMC Bioinform.

[25] Huelsenbeck JP, Ronquist F (2001). MRBAYES: bayesian inference of phylogenetic trees. Bioinformatics.

[26] Hodar C, Zuniga A, Pulgar R, Travisany D, Chacon C, Pino M, Maass A, Cambiazo V (2014). Comparative gene expression analysis of Dtg, a novel target gene of Dpp signaling pathway in the early *Drosophila melanogaster* embryo. Gene.

[27] Kirov N, Childs S, O’Connor M, Rushlow C (1994). The *Drosophila* dorsal morphogen represses the tolloid gene by interacting with a silencer element. Mol Cell Biol.

[28] Darling AE, Mau B, Perna NT (2010). progressiveMauve: multiple genome alignment with gene gain, loss and rearrangement. PLoS ONE.

[29] Khan A, Fornes O, Stigliani A, Gheorghe M, Castro-Mondragon JA, van der Lee R, Bessy A, Cheneby J, Kulkarni SR, Tan G (2018). JASPAR 2018: update of the open-access database of transcription factor binding profiles and its web framework. Nucleic Acids Res.

[30] Papatsenko D (2007). ClusterDraw web server: a tool to identify and visualize clusters of binding motifs for transcription factors. Bioinformatics.

[31] Schneider TD, Stephens RM (1990). Sequence logos: a new way to display consensus sequences. Nucleic Acids Res.

[32] Rushlow C, Levine M (1990). Role of the *zerknullt* gene in dorsal–ventral pattern formation in *Drosophila*. Adv Genet.

[33] Rafiqi AM, Lemke S, Schmidt-Ott U (2010). Postgastrular zen expression is required to develop distinct amniotic and serosal epithelia in the scuttle fly *Megaselia*. Dev Biol.

[34] Ashe HL, Levine M (1999). Local inhibition and long-range enhancement of Dpp signal transduction by Sog. Nature.

[35] Mizutani CM, Nie Q, Wan FY, Zhang YT, Vilmos P, Sousa-Neves R, Bier E, Marsh JL, Lander AD (2005). Formation of the BMP activity gradient in the *Drosophila* embryo. Dev Cell.

[36] Stathopoulos A, Van Drenth M, Erives A, Markstein M, Levine M (2002). Whole-genome analysis of dorsal-ventral patterning in the *Drosophila* embryo. Cell.

[37] Reeves GT, Stathopoulos A (2009). Graded dorsal and differential gene regulation in the *Drosophila* embryo. Cold Spring Harb Perspect Biol.

[38] Rembold M, Ciglar L, Yanez-Cuna JO, Zinzen RP, Girardot C, Jain A, Welte MA, Stark A, Leptin M, Furlong EE (2014). A conserved role for Snail as a potentiator of active transcription. Genes Dev.

[39] Khan A, Fornes O, Stigliani A, Gheorghe M, Castro-Mondragon JA, van der Lee R, Bessy A, Cheneby J, Kulkarni SR, Tan G (2018). JASPAR 2018: update of the open-access database of transcription factor binding profiles and its web framework. Nucleic Acids Res.

[40] Zhang H, Levine M, Ashe HL (2001). Brinker is a sequence-specific transcriptional repressor in the *Drosophila* embryo. Genes Dev.

[41] Kirov N, Zhelnin L, Shah J, Rushlow C (1993). Conversion of a silencer into an enhancer: evidence for a co-repressor in dorsal-mediated repression in *Drosophila*. EMBO J.

[42] Frank LH, Rushlow C (1996). A group of genes required for maintenance of the amnioserosa tissue in *Drosophila*. Development.

[43] Reim I, Lee HH, Frasch M (2003). The T-box-encoding Dorsocross genes function in amnioserosa development and the patterning of the dorsolateral germ band downstream of Dpp. Development.

[44] Yip ML, Lamka ML, Lipshitz HD (1997). Control of germ-band retraction in *Drosophila* by the zinc-finger protein HINDSIGHT. Development.

[45] Herranz H, Morata G (2001). The functions of pannier during *Drosophila* embryogenesis. Development.

[46] Winick J, Abel T, Leonard MW, Michelson AM, Chardon-Loriaux I, Holmgren RA, Maniatis T, Engel JD (1993). A GATA family transcription factor is expressed along the embryonic dorsoventral axis in *Drosophila* melanogaster. Development.

[47] Liberman LM, Stathopoulos A (2009). Design flexibility in *cis*-regulatory control of gene expression: synthetic and comparative evidence. Dev Biol.

[48] Cowden J, Levine M (2002). The Snail repressor positions Notch signaling in the Drosophila embryo. Development.

[49] Wilson MJ, Kenny NJ, Dearden PK (2014). Components of the dorsal-ventral pathway also contribute to anterior-posterior patterning in honeybee embryos (*Apis mellifera*). EvoDevo.

[50] van der Zee M, Stockhammer O, von Levetzow C, Nunes da Fonseca R, Roth S (2006). Sog/Chordin is required for ventral-to-dorsal Dpp/BMP transport and head formation in a short germ insect. Proc Natl Acad Sci USA.

[51] Lilja T, Qi D, Stabell M, Mannervik M (2003). The CBP coactivator functions both upstream and downstream of Dpp/Screw signaling in the early *Drosophila* embryo. Dev Biol.

[52] Jazwinska A, Rushlow C, Roth S (1999). The role of brinker in mediating the graded response to Dpp in early *Drosophila* embryos. Development.

[53] O’Connor MB, Umulis D, Othmer HG, Blair SS (2006). Shaping BMP morphogen gradients in the *Drosophila* embryo and pupal wing. Development.

[54] Heitzler P, Haenlin M, Ramain P, Calleja M, Simpson P (1996). A genetic analysis of pannier, a gene necessary for viability of dorsal tissues and bristle positioning in *Drosophila*. Genetics.

[55] Mirzoyan Z, Pandur P (2013). The Iroquois complex is required in the dorsal mesoderm to ensure normal heart development in *Drosophila*. PLoS ONE.

