# Supplementary figures and images for: The dorsoventral patterning of Musca domestica embryos: insights into BMP/Dpp evolution from the base of the lower cyclorraphan flies
Source: EvoDevo. 2018 May 16;9:13. doi: 10.1186/s13227-018-0102-5 (PMC5956798; doi:10.1186/s13227-018-0102-5)

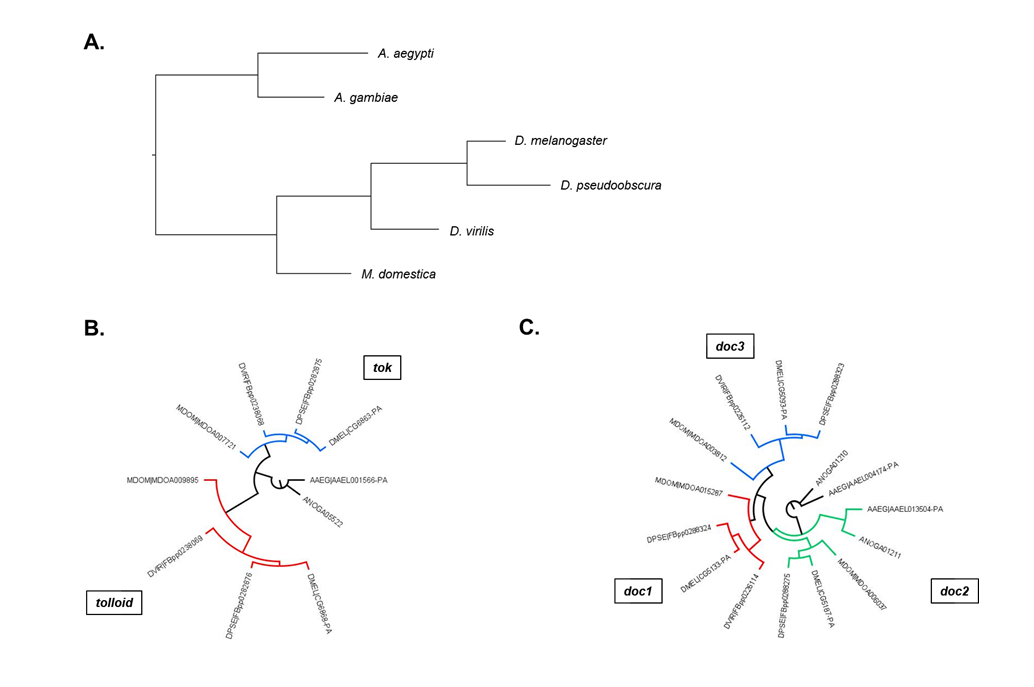

Supplement: Supplementary file 1 — Additional file 1: Fig. S1. Panel (A) shows the UPGMA phylogenetic reconstruction based on the total number of orthologs identified by Proteinortho. Species that have a large part of their proteome in common are grouped. Panels (B) and (C) correspond to phylogenetics trees generated by bayesian approach, based on the alignment of the whole orthologs set for tolloid and Dorsocross proteins, respectively. [file 13227_2018_102_MOESM1_ESM.tif]

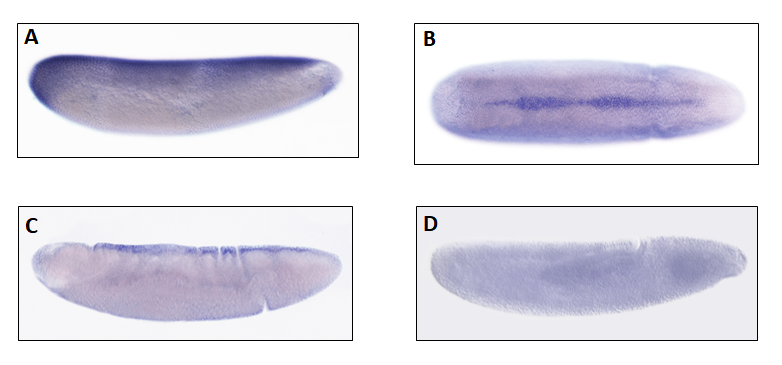

Supplement: Supplementary file 2 — Additional file 2: Fig. S2. Images show the dynamics of the expression, revealed by in situ hybridization, of Md.zen during consecutive stages of embryo development: (A) early cellular blastoderm, (B) late cellular blastoderm at the onset of gastrulation, (C) during gastrula stage and (D) at the end of germ band extension. In all the cases, embryos are oriented from anterior to the left. All images correspond to lateral views except (B) which is a dorsal view. [file 13227_2018_102_MOESM2_ESM.tif]

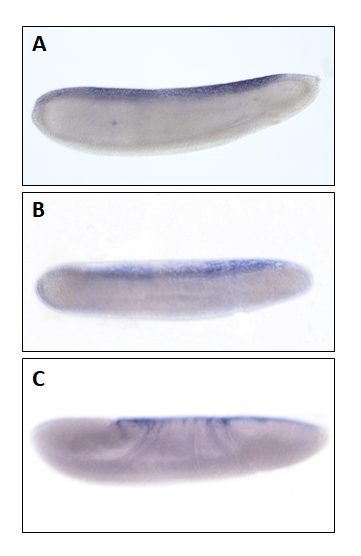

Supplement: Supplementary file 3 — Additional file 3: Fig. S3. Images show the dynamics of the expression, revealed by in situ hybridization, of Md.tld from (A) early cellular blastoderm, the onset of gastrulation (B) and during gastrula stage (C). In all the cases, embryos are oriented with anterior to the left. All images correspond to lateral views except (B) which is a lightly ventrolateral view. [file 13227_2018_102_MOESM3_ESM.tif]
